# Antimicrobial and Gas Barrier Crustaceans and Fungal Chitin-Based Coatings on Biodegradable Bioplastic Films

**DOI:** 10.3390/polym14235211

**Published:** 2022-11-30

**Authors:** Luca Panariello, Maria-Beatrice Coltelli, Ahdi Hadrich, Francesca Braca, Stefano Fiori, Amit Haviv, Filip Miketa, Andrea Lazzeri, Andreas Staebler, Vito Gigante, Patrizia Cinelli

**Affiliations:** 1 Department of Civil and Industrial Engineering, University of Pisa, 56122 Pisa, Italy; 2 National Interuniversity Consortium of Materials Science and Technology (INSTM), 50121 Florence, Italy; 3 Biomass Valorization Platform-Materials, CELABOR s.c.r.l., 4650 Chaineux, Belgium; 4 ARCHA Srl, Via di Tegulaia 10/A, 56121 Pisa, Italy; 5 Condensia Quimica, C/Junqueras 11-A, 08003 Barcelona, Spain; 6 TIPA, 6 HaHarash St., Hod Hasharon 4524079, Israel; 7 Bio-Mi Ltd., Put Brdo 15, 51 211 Matulji, Croatia; 8 Planet Bioplastics s.r.l., Via S. Giovanni Bosco 23, 56017 Pisa, Italy; 9 Fraunhfer-Institute for Process Engineering and Packaging, 85354 Freising, Germany

**Keywords:** coating, bioplastic, biobased, biodegradable, antimicrobial, gas barrier, polyesters, chitin, biopolymer, nanofibrils, shrimp, fungi, mushroom, PLA, PBAT, PBS, PBSA, PHBV

## Abstract

Chitin nanofibrils (CN) can be obtained from crustaceans and fungal sources and can be used for preparing coatings for bioplastic films, that are fundamental for developing a safe and sustainable biodegradable food packaging. Coatings with different concentrations of CN from shrimps were applied on different bioplastic substrates, like Poly (butylene succinate-co-adipate)/Poly(3-hydroxybutyrate-co-3-hydroxyvalerate (PBSA/PHBV) blend, Polybutylene succinate (PBS), and Polybutylene adipate terephthalate/Poly(lactic acid) (PBAT/PLA) blend, but the adhesion to the substrates was scarce. On the contrary, the fungal-based CN showed a better adhesion. Additionally, it was found that the use of an additive based on oligomeric lactic acid was useful to prepare a coating with an improved adhesion to bioplastics. The gas barrier properties to oxygen and water vapour of coated and un-coated films were measured, revealing an improvement of these properties thanks to applied coatings, especially towards the oxygen. Antimicrobial properties and biodegradation capacity were also evaluated revealing an antibacterial effect of the coatings that did not significantly interfere with their biodegradability. The results are discussed and interpreted considering the correlation between composition and macromolecular structures with the observed functional properties.

## 1. Introduction

Biodegradable biobased plastic films are currently used and further developed because they can play a strategic role in green transition [1,2,3,4,5]. In fact, if we consider only their constitutive matter and their end of life, thanks to their renewability, unlike fossil films, they contribute neither to depletion of fossil carbon nor at the increase in CO_2_ concentration in atmosphere [6]. Thanks to their biodegradability, that should be defined in relevant contests, such as industrial composting plants, anaerobic digestion plants, and home-composting, they offer alternatives to landfill and incineration, that represent the worst methodologies for waste management [7]. Moreover, their mechanical or chemical recyclability, yet assessed in some cases [8,9,10], will also prolong their lifecycle, making biobased and biodegradable plastics carbon storage systems up to their final biodegradation at zero emissions. In the COVID-19 period, the consumption of plastic has increased, thus the transition to circular approaches in packaging has become more urgent [11].

Nevertheless, on one hand it is necessary to improve biobased and biodegradable plastic films’ gas barrier properties, considering that generally several bioplastic-based films show a lower barrier to O_2_ than PET and to water vapour than PE or PP [12]; on the other hand, developing functional biobased films with additional desired properties in the packaging sector, such as antimicrobial or antioxidant features, can boost the extension of their market [13] and thus the replacement of fossil-based with renewable packaging.

For these reasons, the surface coating of biodegradable plastic films (in order to improve their gas barrier properties and for conferring antimicrobial features) is a crucial research topic [14]. Gigante et al. [15] have evidenced that biobased liquid coatings based on biopolymers of different natures were mainly used up to now for this purpose.

Chitin and chitosan (poly(N-acetylglucosamine), obtained by the deacetylation of chitin) were widely investigated for their good applicability, being polysaccharidic biopolymers, on cellulosic substrates. The application of chitin nanofibrils (CN), representing the crystalline part of chitin, or chitosan onto cellulosic paperboards resulted in significant improvement of fresh-food shelf-life packed in coated paperboard with respect to an untreated one [16]. Moreover, chitin nanofibrils were also applied to cellulosic tissues by wet coating [17] or by electrospray [18], showing antimicrobial properties.

The antimicrobial action of chitosan is attributed to its capacity to bear positive charges because of protonation of -NH_2_ groups in slightly acidic solutions, where the polymer is soluble, inducing pathogens cell walls disruption [19]. The resulting CNs are insoluble in slightly acidic water solutions, but they behave similarly because they present positive charges on their surface. The high surface-to-volume ratio typical of nanofibrils confer to CN water suspensions high stability because of nanofibrils repulsion, and also the observed antimicrobial properties.

On the contrary, CN incorporated in biopolyester films tested for sanitary applications showed only an indirect antimicrobial action due to its immunomodulatory activity towards skin cells [20]. It was hypothesized that, in these films, chitin nanofibrils are mainly embedded in the biopolyester matrix and they are not available on the bioplastic surface. Thus, they cannot exert their antibacterial action. These results indicated that developing a coating to be applied on the surface of bioplastic films could be much more promising to achieve antimicrobicity.

In the field of food preservation, the application of chitosan as an antimicrobial in food packaging was widely investigated, as reviewed by Kumar et al. [21]. Edible coatings for fruits and vegetables were obtained by several authors [22]. Pagno et al. evidenced that the nutraceutical quality of tomato fruit was influenced by the presence of a chitosan coating [23]. Some researchers described the usefulness of a chitosan edible coating on postharvest life and quality attributes of kiwifruit [24], some others evidenced the increase in shelf life of sliced mango fruit [25]. La et al. [26] proposed a coating consisting of chitosan and ZnO nanoparticles for the postharvest phase of banana fruits. Other researchers suggested the possible application of chitosan-based edible coatings to cheese [27]. Thus, foods with different surface properties were considered.

Regarding plastic and bioplastic films, many scientific papers can be found in the literature regarding chitosan [21]. It was applied onto polyethylene films by casting from acetic acid solutions, resulting in an improved gas barrier to oxygen [28]. A crucial issue was the adhesion between the chitosan film and polyethylene, so that a preliminary dielectric barrier discharge (DBD) plasma treatment of polyethylene was found as a good methodology to increase it [29]. Poly(lactic acid) (PLA) films were also modified by applying a squid chitosan coating prepared in hydrochloric acid. The PLA was preliminarily compounded with 20% of glycerol and processed by blown film extrusion. The bilayer film was used for increasing the shelf life of vegetables, but the results were controversial. The authors considered that the acidic layer of chitosan could induce some degradation of the PLA film [30]. To increase the interaction between the PLA-based layer and the chitosan dip coating Hongsriphan and Sanga [31] treated the film by corona discharge. Gartner et al. [32] applied a liquid coating consisting of PLA (previously dissolved in acetone and additivated with methylene diphenyl diisocianate) and chitosan onto PLA films. Hence, they formulated a coating containing PLA but also a fossil reactive additive (methyldiphenyl diisocyanate) to enhance its adhesion to the PLA substrate.

In the present paper, the formulation and application of fully biobased CN-based coatings on different biobased films is carried out. Both chitin nanofibrils obtained from shrimps and fungi were considered and differences in their features and properties of the coating were evidenced through SEM and ATR-IR analysis of the raw materials while their affinity to biopolymers was evaluated by the analysis of covered surface and tape test. Antibacterial tests were carried out and the results discussed considering their potential negative effect on biodegradability. Oxygen and water vapour barrier properties were also evaluated through contact angle measurement to evidence the effect of coatings on the permeability of gas.

## 2. Materials and Methods

### 2.1. Materials

Partially deacetylated chitin nanofibrils (CNs) suspensions were supplied by Celabor (Chaineux, Belgium). The suspensions were obtained through a chemical pre-treatment followed by a mechanical defibrillation process using an ultra-fine friction grinder Super masscolloider (Masuko^®^ Sangyo Co. Ltd., Kawaguchi, Japan) equipped with two ceramic nonporous grinders adjustable at any clearance between the upper and lower grinder. Chemical pre-treatment was performed by a partial deacetylation of commercial chitin with fungal and crustacean origins (both purchased from Glentham Life Sciences Ltd., Corsham, UK) using concentrated sodium hydroxide. The reaction was stopped when a degree of deacetylation (DDA) of 16% was reached. The product was then purified until the pH value reached 6.5~7. After the partial deacetylation, the resultant chitin suspensions were then prepared for mechanical defibrillation by dispersing them in acidified water at different concentration and grinding time. The dispersions were then manually poured into the grinder and the partially deacetylated chitin suspensions fed into the hopper were dispersed by centrifugal force into the clearance between the grinding stones, where they were ground into ultra-fine particles, after being subjected to massive compression, shearing, and rolling friction forces. CNs were thus obtained and stored at 4 °C until further use. CNs dispersions were used as produced.

Oligomeric lactic acid (OLA) with a Mw = 1350 Da was provided by Condensia Quimica (Barcelona, Spain) and was used as received. Formulations containing OLA were prepared dissolving 0.5 g of OLA in ethanol (96%, Carlo Erba Reagents S.r.l., Cornaredo, Italy) for each gram of dry chitin mass. After complete dissolution of OLA, the chitin dispersion was added to the OLA solution. The amount of ethanol was measured considering that the final formulation must have a water to ethanol mass ratio of 0.5. As a 1.5% chitin was used for all the formulations containing OLA it was possible to calculate the final concentration of OLA and CN that were, respectively, 0.33% and 0.75%.

Substrates were chosen from a selection of industrially relevant commercial bioplastics formulations. PBSA/PHBV-based, PBS-based and PBAT/PLA-based films were selected to embrace a wide range of biopolymeric substrates, with characteristics that can be found in the literature [33,34,35]. Specifically, the selected films are suitable for packaging because of their good transparency and flexibility. They show a tensile strength in the range 25–35 MPa and an elongation at break in the range 200–400%. The films were provided by producers participating at the work: Bio-mi Ltd. (Put Brdo, Croatia), Planet Bioplastic s.r.l. (Pisa, Italy) and TIPA (Hod Hasharon, Israel).

### 2.2. Coating Application and Evaluation

All the suspensions were applied on three sheets for each bioplastic substrates with an automatic applicator Zehntner ZAA 2300 (Sissach, Switzerland) equipped with a universal applicator Zehntner Zua 2000 (Sissach, Switzerland). According to a previous work [16] the application speed was set to 10 mm/sec, the width of applicator was 20 cm and the thickness was set to 150 µm. Homogeneity of coating application was based on the analysis of pictures acquired on the dried coated films. From the images the covered surface ratio was evaluated defined as the percentage of surface covered by the treatment respect to the total surface of the substrate. The value was calculated with the software Image J (ImageJ, National Institutes of Health, WI, USA, version 1.52n)) applied on at least 5 high resolution pictures recorded on coated surfaces having dimensions 50 × 50 mm. Mean values with their standard deviation were calculated for each sample. The presence of adhesion was also assessed on three coated points of the film, through tape test according to the ASTM D3359, test method A. Statistical analysis was carried out on covered surface ratio values of all the coated films to discuss the significance of the differences. Two set of data were taken in account; the first considered the different pure CN formulation applied on PBSA/PHBV substrate while the second one regarded the application of CN, with and without OLA, on all the considered films. A Tukey test was performed with a Minitab software (Minitab Ltd., Coventry, UK, version 19.2020.1) setting a significance threshold of 0.05. Samples were grouped according to Tukey method with a 95% of confidence. Samples that do not share a letter indicate that there is less than 5% probability that the results are not significantly different.

### 2.3. Infrared Spectroscopy

Chitin powder and CN suspensions from different sources were analysed by infrared spectroscopy using a Nicolet T380Thermo Scientific instrument equipped with a Smart ITX ATR accessory with a diamond plate (Thermo Fisher Scientific, Waltham, MA, USA). Spectra were acquired in the range 4000–500 cm^−1^, collecting 256 scans at 4 cm^−1^ resolutions. Both powders and suspensions were analysed as received pouring an amount of sample to completely cover the surface of the ATR crystal. Two spectra for each sample were acquired. The most representative and with a better signal-to noise spectrum was inserted in the figures.

### 2.4. Scanning Electron Microscopy (SEM)

Chitin powders and suspensions were observed under a field emission scanning electron microscopy (FESEM), FEG-Quanta 450 instrument (FEI, Hillsboro, OR, USA) to observe the effectiveness of the defibrillation process. Powders were mounted directly on SEM stubs while suspensions were prepared as follow. A drop of diluted chitin suspension (1:1000 *v/v* in water) was poured on a microscopy slide and dried at room temperature. The slide was mounted on a SEM stub and observed under the microscope using a voltage 15 kV at different magnification (mainly 30,000× and 120,000×). The surface of coated and uncoated substrates was also compared under the SEM to evaluate the morphological changes using a voltage 10 kV at different magnification (mainly 60,000×). Two stubs were prepared for each sample. A list of the studied substrates and suspensions was reported in Table 1.

### 2.5. Biodegradation Test

#### 2.5.1. Industrial Biodegradation Test

Industrial biodegradation test was performed according to ISO 14855-1 applied at 58 ± 2 °C. According to EN 13,432 standard, the material can be considered biodegradable under industrial composting conditions if at least 90% (as absolute or relative value, referred to a suitable reference substance) after a constant level of biodegradation has been achieved for both the test material and the reference substance within 6 months. Biodegradability tests were performed in bioreactors (composting vessels) composed by one test substance, one blank, and one positive control, all in three replicates.

#### 2.5.2. Home Biodegradation Test

Home biodegradation was tested according to ISO 14855-1 applied at ambient temperature (between 20 °C and 30 °C). The temperature must be kept below 30 °C for the duration of the test. The required percentage of biodegradation is specified in the Ok compost HOME document: Initial acceptance tests defined by TUV Austria [36]. In particular, a material can be considered home biodegradable if more than 90% of the original dry mass of test materials degrades within a maximum period of 12 months. Biodegradability tests were performed in bioreactors (composting vessels) composed by one test substance, one blank and one positive control, all in three replicates.

### 2.6. Barrier and Surface Analysis

Oxygen transmission rate (OTR) was determined according to DIN 53380-3 at 23 °C and 50% relative humidity. The water vapour transmission rate (WVTR) was determined according to DIN 53122-1 at 23 °C and relative humidity of 85% to 0%. For each sample at least three replicates were performed. Mean values with their standard deviation were calculated for each sample.

The determination of the free surface energy of solid surfaces was performed by measuring the contact angle technique by distinguish the dispersive and the polar components of surface energy as reported in DIN 55660-2:2011-12 while the evaluation of the data was performed with the Owens–Wendt–Rabel and Kälble (OWRK) evaluation. For each sample at least three replicates were performed. Mean values with their standard deviation were calculated for each sample.

Statistical analysis was carried out to discuss the significance of the differences observed between the OTR, WVTR and free surface energy of considered samples. Two-sample t-test analyses were performed with a Minitab software (Minitab Ltd., Coventry, UK, v 19.2020.1) setting a significance threshold of 0.05. *p*-values obtained from the statistical analysis were reported for each considered couples. A *p*-value less than 0.05 indicates that there is less than 5% probability that the results are not significantly different.

### 2.7. Antimicrobial Test

Tests were performed on two bacterial species, *Escherichia coli* and *Staphylococcus aureus*, following the standard method ISO 22196:2011 (at 5 × 10^5^ cells/mL, as stated by the method) on flat specimen (50 × 50 mm) of coated and uncoated substrates.

When the test was deemed valid, the antibacterial activity was calculated according to Equation (1).

R = (U_t_ − U_0_) − (A_t_ − U_0_) = U_t_ − A_t_ (1)

where R is the antibacterial activity, U_0_ is the average of the common logarithm (base 10 logarithm) of the number of viable bacteria, in cells/cm^2^, recovered from the untreated test specimens immediately after inoculation, U_t_ is the average of the common logarithm of the number of viable bacteria, in cells/cm^2^, recovered from the untreated test specimens after 24 h, and A_t_ is the average of the common logarithm of the number of viable bacteria, in cells/cm^2^, recovered from the treated test specimens after 24 h.

The value of the antibacterial activity can be used to evaluate the effectiveness of an antibacterial agent or treatment. According to ISO 22196:2011 “the antibacterial-activity values used to define the effectiveness shall be agreed upon by all interested parties”.

## 3. Results

### 3.1. SEM Characterization of Shrimp Chitin

CN dispersions at different concentration and defibrillation time were prepared starting from the chitin shrimp powder. Both powder and suspensions were examined by SEM to investigate the effect of preparation process on the chitin nanofibrils morphology (Table 2).

The not defibrillated chitin powder revealed dimension of hundreds of microns and the typical microfibrillar structure showing also some porosity, typical of the organism from which it was extracted (shrimps) [37]. The higher magnification revealed the fibrillar nanostructure typical of chitin from crustaceans’ exoskeletons. The analysis was performed on the samples obtained by drying the aqueous CN showed the typical nanofibrillated chitin structure. Formation of CN aggregates was caused by the drying process while the presence of spherical particles can be attributed to inorganic salt used during the defibrillation process. No significant differences were noticed changing time and concentration and all the dispersions appeared well defibrillated.

### 3.2. ATR-IR Analysis of Shrimp Chitin

**Figure 1:**
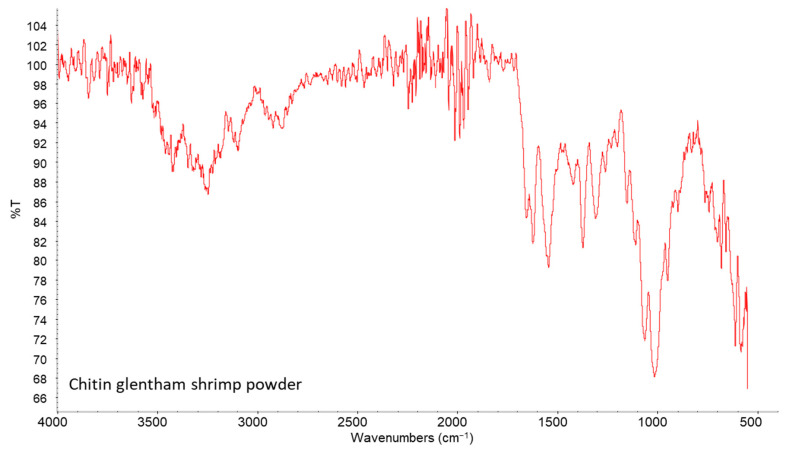
ATR-IR spectrum of shrimp chitin powder.

**Figure 2:**
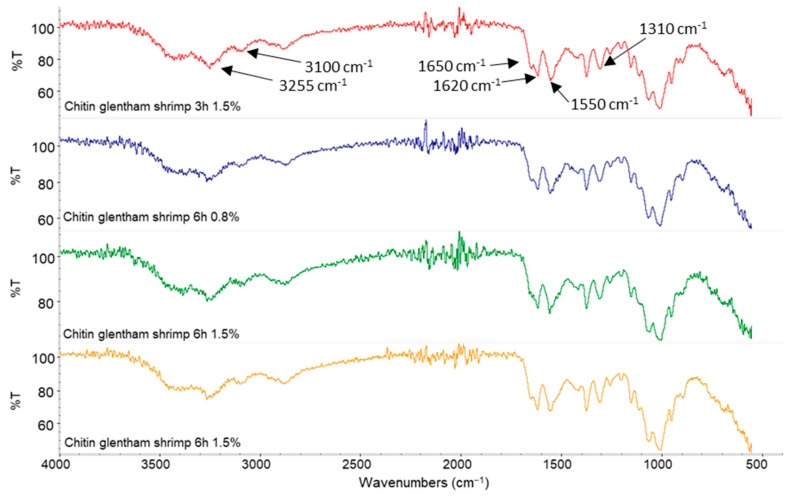
ATR-IR spectra of shrimp CN obtained at different time and concentration.

Chitin powder and suspensions produced at different time and concentration were analysed through ATR-IR spectroscopy (Figure 1 and Figure 2) to investigate differences in the chemical groups/bonds induced by the defibrillation process.

Characteristic signals of chitin amide functional group were represented by the C-N stretching of amide group (amide III) at 1310 cm^−1^, N-H bending of amide group (amide II) at 1550 cm^−1^ and the C-O stretching of amide group (amide I) at 1650 cm^−1^. In the spectra typical signals of chitosan amine group were also showed such as the N-H bending of primary amines at 1620 cm^−1^ symmetric and asymmetric stretching of N-H groups, respectively, at 3100 cm^−1^ and 3255 cm^−1^. No significant differences can be observed between spectra at different time and concentration and the powder of chitin but the presence of both amide and amine signals confirm the nature of partially deacetylated CN as declared by the producer.

### 3.3. Surface Analysis of Plastic Substrates with Chitin Shrimp Suspensions

Chitin nanofibrils dispersions were applied on a film of PBSA/PHBV to compare the affinity of different coatings with a same reference film. Results of covered surface ratio and adhesion were reported in Table 3.

All the substrates showed a low surface affinity with the suspension, that led to the formation of non-continuous films (covered surface ratio < 100%) and no adhesion of the coating to the substrate. However, the evaluation of covered surface ratio evidence an increase in coating-substrate affinity increasing both concentration and processing time.

### 3.4. SEM Characterization of Fungal Chitin

In addition to shrimp, it was reported that chitin can be extracted from different sources such as insects [38] or fungi [39]. The use of a chitin from a different source can be a valid alternative to shrimp chitin to improve its affinity with a plastic substrate. In particular, it was reported that in the chitin from fungi complexes between fungal chitin and glucans are present that can decrease the hydrophilicity of chitin derivatives [40,41,42]. A commercial type of fungal chitin was used as raw material for the production of dispersion and their application on PBSA/PHBV film.

**Figure 3:**
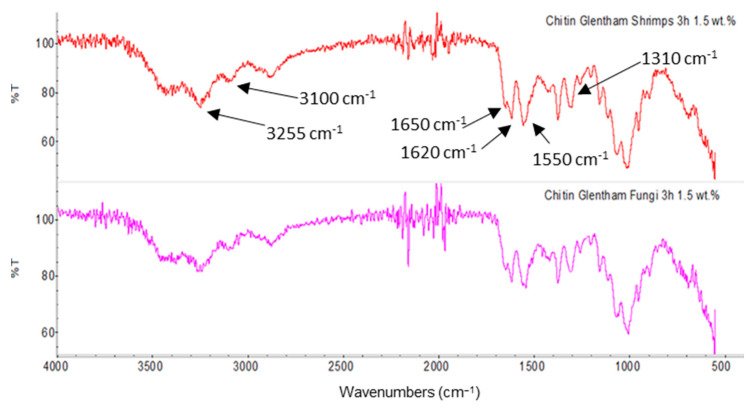
Comparison between ATR-IR spectra of chitin produced by shrimp and fungi.

Moreover, for the fungal chitin, SEM (Table 4) and ATR-IR (Figure 3) analysis was performed as well as the evaluation of the application on PBSA/PHBV film (Table 5).

Both ATR-IR spectra reported in Figure 3, showed the typical amide (1310 cm^−1^, 1550 cm^−1^ and 1650 cm^−1^) and amine signals (1620 cm^−1^, 3100 cm^−1^ and 3255 cm^−1^). Infrared spectroscopy did not evidence any substantial differences between spectra showing that the different source did not significantly affect the chemical structure of the chitin.

The application of chitin dispersion from fungi showed a better wettability of PBSA/PHBV substrate respect to chitin from shrimps reaching a 100% covered surface ratio and a good adhesion.

Statistical analysis of covered surface ratio for the CN coating (both from fungi and shrimp) applied on PBSA/PHBV was reported in Table 6.

Statistical analysis of different covered surface ratio reported in Table 6 revealed that the CN shrimp 6 h 1.5%, CN shrimp 6 h 2.5% and CN fungi 3 h 1.5% coatings cannot be considered significantly different. Consequently, these results evidenced that the 1.5% is the limit concentration to reach the maximum coverage of the PBSA/PHBV surface. CN from fungi required lower concentration and grinding time to reach the same results. Instead, the application of CN shrimp at lower concentration and grinding time resulted in significantly different data, meaning that their coverage capacity was limited.

### 3.5. CN + OLA

In order to increase the affinity of shrimp chitin for the PBSA/PHBV plastic substrate, OLA was added to the formulation as compatibilizer/primer.

**Figure 4:**
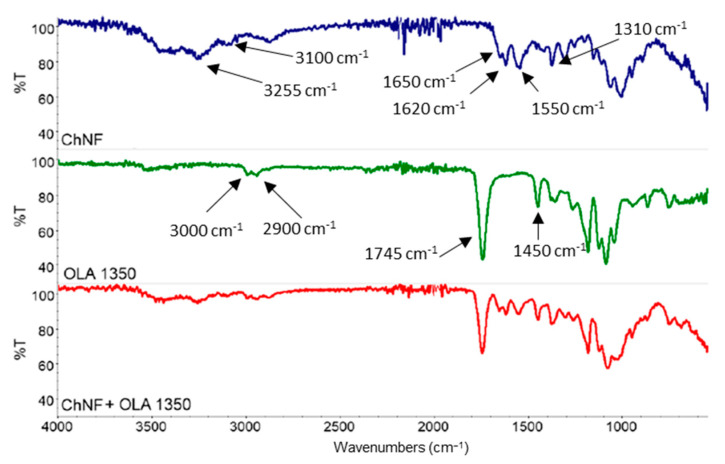
FTIR spectra of CN (shrimps) + OLA 1350 dispersion and single components.

To investigate the effect of OLA on the chemical structure, a comparison between ATR-IR of final formulation and their single components was reported in Figure 4.

OLA showed a typical signal of polyester that includes the stretching of ester carbonyl (C = O) at 1745 cm^−1^, signals relative to CH_2_ and CH_3_ stretching in the range 2900–3000 cm^−1^ and relative bending at 1450 cm^−1^ and many signals in the range 1000–1200 cm^−1^ attributable to different movement of C-O-C bond. Spectrum of the final formulation is a mere superposition of two spectra (CN and OLA) where can be found signals typical of both components. No chemical reaction between OLA and CN seemed to take place.

In Table 7 the results of covered surface ratio and adhesion evaluation on coated films were reported. The effect of OLA with respect to formulation based on the same batch of CN dispersion can thus be deducted.

Statistical analysis of the covered surface ratio for the CN coating, with and without OLA, applied on different substrate is reported in Table 7, grouping the data with letters (statistically different means were represented by a different letter). The analysis confirmed that the addition of OLA to the formulation resulted in a significantly different ability to cover the surface of the films. Moreover, as all the formulation with OLA and all the formulation without OLA were grouped under the same letter, it was possible to deduced that the formulations had the same coverage ability independently by the substrates.

Pure shrimp chitin coating applied on the selected polyester films showed a poor affinity for all of them reaching covered surface ratio values lower of 70% and poor adhesion. Addition of OLA to the formulations increase in all the samples the covered surface ratio to 100% (continuous film) revealing a good adhesion.

SEM micrographs of coated and uncoated substrates were reported in Table 8.

### 3.6. Barrier Properties

**Figure 5:**
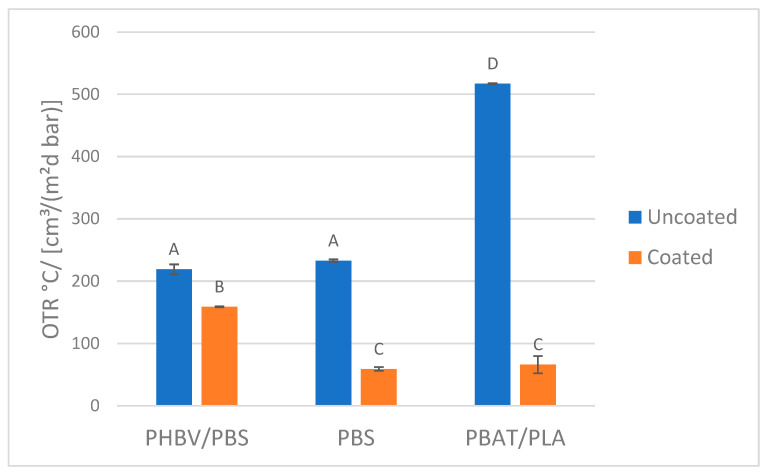
OTR values of coated and uncoated films. The same letter on the column means that values were not significantly different (confidence level 95%).

**Figure 6:**
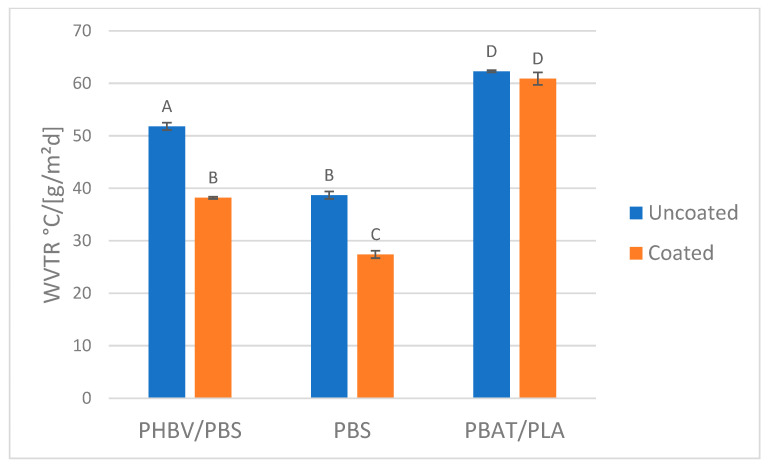
WVTR values of coated and uncoated films. The same letter on the column means that values were not significantly different (confidence level 95%).

Kwaldia et al. [43] reported the production of a polysaccharide/protein system consisting of chitosan and caseinate bilayer. This coating was applied on paper packaging, not on bioplastics films, resulting in an improvement of water vapor barrier properties without any detrimental effect on mechanical properties. Stam et al. [44] performed the layer-by-layer spray coating of cationic CN and anionic cellulose nanocrystal (CNC) suspensions onto poly(lactic acid) (PLA) films. However, multilayer CN/CNC coatings were found to have lower O_2_ permeability and lower haze than those coated with CN or CNCs alone (72% and 86% lower haze, respectively), pointing to a synergistic effect. Interestingly, the same authors showed that the addition of CN to CNC was a promising tool to control mechanical and barrier properties of CNC-based films [45]. Instead, Yu et Al. reported that chitin/chitosan or their combination can be a sustainable choice as barrier agent [46]. Regarding chitin nanofibrils, they can be obtained from several sources: crustaceans, mushrooms, insects (α-chitin) [38] or cocoon and squid pens (β-chitin) [47]. Fan et al. [48] compared cast films from chitin nanofibrils from crab shells (α-chitin) with chitin nanofibrils from squid pens and evidenced that mechanical properties were different but the barrier properties to oxygen were similar and much higher than those of PLA films. In general, comparisons between chitin nanofibrils coming from different sources regarding their physical-chemical properties are quite rare in the scientific literature [49]. In particular, fungal CN showed a better affinity to the plastic substrates. It is reasonable to affirm that preparation of formulation based on fungal CN containing OLA is the best choice to maximise the homogeneity of the coating and consequently their functional properties. Moreover, in the literature it was reported that fungal chitin has a lower hydrophobicity respect to crustacean chitin which made it more suitable as gas barrier [42]. The permeability to oxygen (OTR) and water vapour (WVTR) of the coating based on fungal CN and OLA were, respectively, reported in Figure 5 and Figure 6.

Histograms, reported in Figure 5 evidenced a considerable effect of chitin + OLA coating on the oxygen and water vapour barrier of all the films except for a negligible effect on the WVTR of PBAT/PLA film. As reported in the literature, high crystallinity and strong inter- and intra-sheet hydrogen bonding of chitin chains [46] are reflected in increased barrier properties. Although the fungi CN was chosen for the barrier property tests, it was reported that the shrimp CN can also be successfully used as gas barrier agent [45,50,51].

The effect of coating onto WVTR is not much pronounced, but significant especially for PHBV/PBS and PBS films. The observed WVTR values are certainly lower than the one observed for poly(ethylene terephthalate) (PET) [52,53]. The OTR values of uncoated films are significantly lower than that of poly(ethylene) (PE), poly(propylene) (PP) and poly(styrene) (PS), but higher than the one of PET [54]. The coating determines a significant decrease in OTR, making films competitive with those made with fossil PET. Thus, combining biobased films with this CN-based functional coating actually represents a strategy for replacing this fossil polymer with renewable ones.

Statistical analysis confirmed that the application of a coating results in significantly lower values of OTR for all the substrates and that the oxygen barrier of the raw PBAT/PLA film was significantly lower respect to the other two films. Regarding the WVTR the statistical analysis confirmed that the coating was not able to significantly increase the water vapour barrier of PBAT/PLA film while revealed an effectiveness for the other two films. Instead, considering the uncoated films, their WVTR were all significantly different, confirming the PBS as the film with the best water vapour barrier.

### 3.7. Surface Energy

Surface properties of the coated and uncoated films were measured, and the results were reported in Table 9. The application of the coating determined an increase in Surface Energy of bioplastic films, that mainly impacted the polar component.

Statistical analysis was performed separately on surface energy and its single component and reported as letters on the mean data (statistically different means were represented by a different letter). Letters reported on the surface energy were all different meaning that the surface energy values of the coated and uncoated substrates were all significantly different between them. The same comment can be reserved to results of statistical analysis performed on the polar part, while the dispersive part showed a different behaviour for the PHBV/PBS films. In fact, in this type of film, the coating process did not induce a significantly change in the dispersive part.

### 3.8. Antibacterial Test

CN or chitosan were commonly used for their antimicrobial properties [16,55,56,57] often mixed with other components. For instance Panariello et al. [56] developed a spray coating consisting of CN, CN complexed with nanolignin and the latter containing Vitamin E. This coating was applied by spray onto poly(hydroxyalcanoate)/starch biobased flexible films and tested as active system for cosmetic application (beauty masks), for exploiting the antimicrobial and skin regenerative properties of CN.

The addition of OLA in the chitin-based formulation could compromise or enhance these properties. In order to investigate this effect, the antibacterial properties of different coatings, with and without OLA, were tested and compared with the uncoated films. Moreover, the antibacterial properties of the OLA without chitin were also considered. All the results are reported in Table 10.

Antibacterial activity, measured as the difference between the normal log of the treated and untreated values, could give an indication about the presence and magnitude of antibacterial properties.

Statistical analysis was performed on results of different coated films compared with the uncoated ones. Regarding the CN fungi formulation, the addition of OLA significantly affected antibacterial properties of the treatment while the formulation without OLA can improve the antibacterial effect only towards *S. aureus*. Moreover, comparing the effect of CN + OLA from different sources, it was evident that both can significantly reduce the bacterial proliferation. Comparing the antibacterial properties of each plastic substrates coated with CN shrimp 3 h 1.5% + OLA with the respective uncoated films, a significant increment of antibacterial effect was reported in all the coated films except for the PLA/PBAT + CN shrimp 3 h 1.5% + OLA, whose effect towards *S. aureus* can be statistically confused with the one of the pure PBS. Lastly, it was confirmed that the results obtained by using OLA are significantly different respect to untreated substrates.

### 3.9. Biodegradation Test

Blends such as PBAT/PLA and PBSA/PHBV are well-known biodegradable polymers [58,59,60]. However, PLA/PBAT-based blends are known to be compostable in industrial environments [61,62], whereas PHBV and PBSA resulted home compostable [63,64,65]. Thus, home compostability tests were performed on the coated PHBV/PBSA film, whereas industrial compostability was tested onto coated PLA/PBAT films. These tests are significant to control if the application of coating with antibacterial properties (as reported in Table 10) can delay the time necessary for the biodegradation in industrial or home composting.

According to the results of biodegradability tests reported in Table 11, both the PBAT/PLA and PBSA/PHBV film coated with chitin +OLA resulted suitable for, respectively, the industrial (>90% in 6 months at 58 ± 2 °C) and the home (>90% in 12 months at room temperature) composting. The general biodegradation behaviour of uncoated films was thus not altered by the coating.

## 4. Discussion

Chitin prepared under different concentration and mechanical defibrillation time were characterized with SEM and ATR-IR. Although the morphology and chemical structure resulted similar, their application on a plastic substrate evidenced an enhanced affinity to the substrate and an improved homogeneity of coatings by increasing the CN concentration, probably because of their improved capability of forming a continuous film on the surface. Moreover, a significant impact of mechanical defibrillation time was also noticed, indicating that applying a higher mechanical stress during defibrillation resulted in a better homogeneity of the films.

Comparing SEM micrographs from fungal source with those relative to shrimp chitin in Table 2 it was evident an higher order in the fungal chitin, indicating a slightly less efficient defibrillation, and the presence of submicrometric bundles, as also reported in the literature [49]. Nevertheless ATR-IR results did not evidence significant chemical differences and chitin nanofibrils from fungal sources were successfully obtained. A substantial difference between the chitin from different sources was represented by their affinity to the PBSA/PHBV substrate, revealing a higher affinity to it of fungal chitin than shrimps one, that can be attributed at its slightly lower polarity. In fact in fungi chitin is linked to glucans [66]. So, the surface deacetylation occurred during production of CN can result in less positively charged chitin nanofibrils because the glucans, having not amide groups such as chitin, are not modified in a positively charged polymer by the deacetylation. This can be linked also at the higher dimension observed for fungal CN, as the repulsion due to superficial -NH^3+^ groups can be beneficial in deagglomerating nanofibrils.

Experimental results reported in Table 7 reveal that OLA can be an excellent additive to increase the affinity of shrimp chitin for the PBSA/PHBV substrate, achieving a covered surface ratio of about 100%. Moreover, it proved to be an excellent compatibilizer not only for the PBSA/PHBV but also for the other polyester films based on PBS and PBAT/PLA. From the comparison between micrographs of coated and uncoated films it was visible the presence in the coated samples of a superficial film with nanofibrils entangled inside. In all the micrographs it was possible to observe the surface of the uncoated polymer through the coating. Since it is reported in the literature that for carbon-based materials the emission of secondary electrons occurs approximately in the first 50 nm of the sample [67], it is reasonable to think at the formation of a very thin film. This innovative coating formulation was also shown to improve the oxygen and water-vapour barrier on almost all the selected substrates. Analysis of surface energy evidenced that uncoated films were characterized by a similar (but significantly different) surface energy with a poor polar part respect to the dispersive one. Application of a coating onto the surface of films results of an increase in surface energy in all of them. In particular, the polar part underwent a significant increase while the dispersive part showed limited changes due to the application of the coating. The treatment application has therefore made the films more available towards polar-type interactions without compromising dispersive-type interactions (such as van der Waals forces). The coating can thus behave better with polar additional treatments, such as inks or adhesives, generally used in packaging. The natural antibacterial activity was not contrasted by the presence of OLA which, on the contrary, contributed to improve the antibacterial efficacy. In fact, the results in Table 10 showed that the addition of OLA in the formulation strongly increases the antimicrobial properties of the coating. Comparing the antimicrobial effect of fungal chitin respect to the shrimp chitin, it was clear that both revealed a significant antibacterial activity. Moreover, it was demonstrated that the formulation with OLA was effective on all the considered substrates. Lastly, it was also shown that the OLA has not antibacterial properties but is only a good additive for the chitin, being a compatibilizer between the polar CN and the less polar biopolyesters substrates. The increase in antibacterial efficacy of the formulation containing OLA can be then attributed to a better dispersion of the chitin nanofibrils in the coating, making them more available on the surface, where the antimicrobial action is exerted. Biodegradation test demonstrated that the application of an antibacterial coating on plastic substrates did not significantly inhibit their degradation in both industrial and home composting conditions.

## 5. Conclusions

CN suspensions coming from fungi and shrimps, were applied on commercial bioplastic substrates based on PBSA/PHBV, PBS and PLA/PBAT, representative of home or industrial compostable biobased films, recently introduced on the market. The combination with commercial oligomeric lactic acid resulted in a successful strategy, improving the homogeneity and adhesion as well as the antimicrobial and gas barrier properties of coatings. Biodegradation behaviour of films was not altered by the presence of the coating. The developed coatings are biobased (produced from waste of sea food or mushrooms cultivation), biodegradable and contributed to produce high-performance bioplastics. This work can represent a concrete answer to the general request for availability of environmentally friendly packaging, integrating the necessity to make a more extensive use of renewable resources and to apply circular economy principles.

## Figures and Tables

**Table 1:** List of substrates and suspensions.

Substrates	Suspensions
PBSA/PHBV	CN shrimp 3 h, 1.5 wt%
PBS	CN shrimp 6 h, 1.5 wt%
PBAT/PLA	CN shrimp 6 h, 2.5 wt%
	CN shrimp 6 h, 0.8 wt%
	CN fungi 3 h, 1.5 wt%

**Table 2:** Micrographs of chitin powder and CN suspensions from shrimps at different time and concentration.

Sample	Micrographs	
Chitin shrimp powder	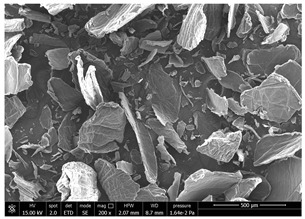	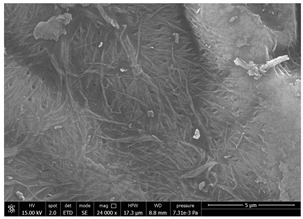
CN shrimp 3 h, 1.5 wt%	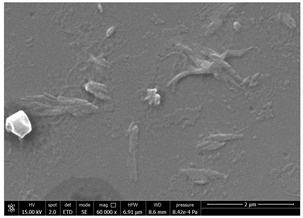	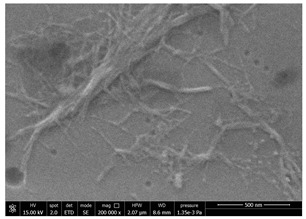
CN shrimp 6 h, 1.5 wt%	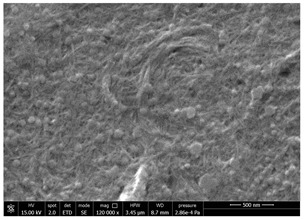	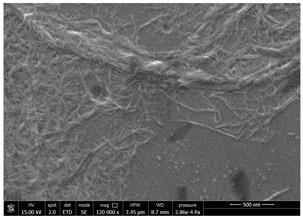
CN shrimp 6 h, 2.5 wt%	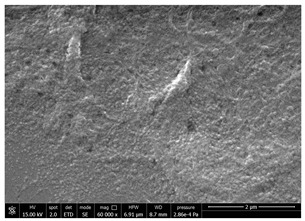	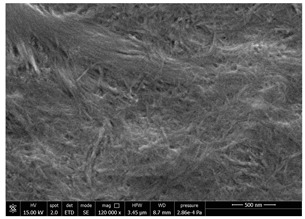
CN shrimp 6 h, 0.8 wt%	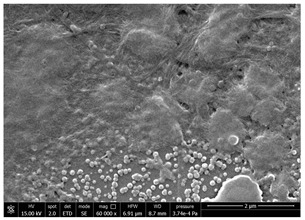	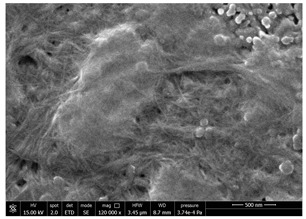

**Table 3:** Surface analysis of PBSA/PHBV films coated with chitin from shrimps at different time and concentration.

	Covered Surface Ratio (%)	Adhesion
PBSA/PHBV + CN shrimp 3 h 1.5%	69 ± 9	1 A Removal from most of the area of the X under the tape
PBSA/PHBV + CN shrimp 6 h 0.8%	78 ± 4	1 A Removal from most of the area of the X under the tape
PBSA/PHBV + CN shrimp 6 h 1.5%	86 ± 8	1 A Removal from most of the area of the X under the tape
PBSA/PHBV + CN shrimp 6 h 2.5%	96 ± 3	1 A Removal from most of the area of the X under the tape

**Table 4:** Micrographs of chitin suspension from fungi.

Sample	Micrographs	
Chitin fungi powder	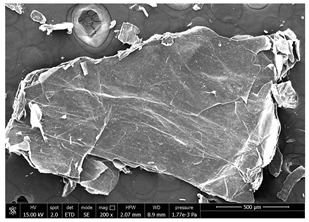	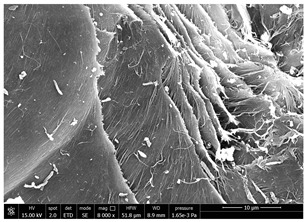
CN fungi 3 h, 1.5 wt%	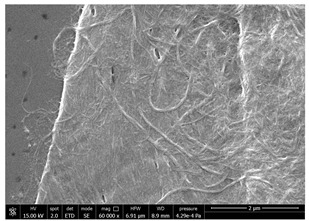	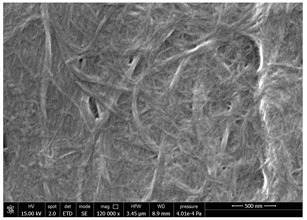

**Table 5:** Surface analysis of PBSA/PHBV films coated with chitin from fungi.

	Covered Surface Ratio (%)	Adhesion
PBSA/PHBV + CN fungi 3 h 1.5%	99 ± 1	5 A no peeling or removal

**Table 6:** Statistical analysis on Covered surface ratio results. The same letter means that values were not significantly different (confidence level 95%).

Sample	Covered Surface Ratio (%)
PBSA/PHBV + CN shrimp 3 h 1.5%	69 ± 9 ^a^
PBSA/PHBV + CN shrimp 6 h 0.8%	78 ± 4 ^a,b^
PBSA/PHBV + CN shrimp 6 h 1.5%	86 ± 8 ^b,c^
PBSA/PHBV + CN shrimp 6 h 2.5%	96 ± 3 ^c^
PBSA/PHBV + CN fungi 3 h 1.5%	99 ± 1 ^c^

**Table 7:** Surface analysis of different polyester films coated with chitin from shrimps with and without the addition of OLA. The same letter means that values were not significantly different (confidence level 95%).

	Covered Surface Ratio (%)	Adhesion
PBSA/PHBV + CN shrimp 3 h 1.5%	69 ± 9 ^a^	1 A Removal from most of the area of the X under the tape
PBSA/PHBV + CN shrimp 3 h 1.5% + OLA	99 ± 1 ^b^	5 A no peeling or removal
PBS + CN shrimp 3 h 1.5%	63 ±6 ^a^	4 A trace peeling or removal along incision or at their intersection
PBS + CN shrimp 3 h 1.5% + OLA	98 ± 2 ^b^	5 A no peeling or removal
PBAT/PLA + CN shrimp 3 h 1.5%	58 ±5 ^a^	4 A trace peeling or removal along incision or at their intersection
PBAT/PLA + CN shrimp 3 h 1.5% + OLA	99 ±1 ^b^	5 A no peeling or removal

**Table 8:** Micrographs of coated and uncoated substrates.

Sample	Micrographs	
	Coated	Uncoated
PHBV/PBS + CN shrimp 3 h 1.5% + OLA	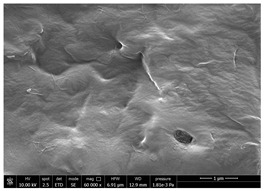	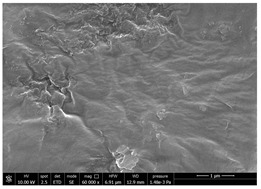
PBS + CN shrimp 3 h 1.5% + OLA	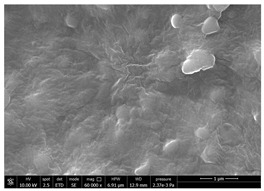	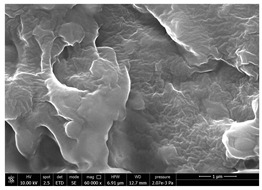
PBAT/PLA + CN shrimp 3 h 1.5% + OLA	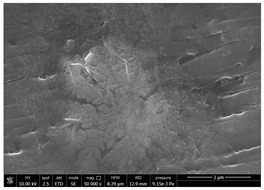	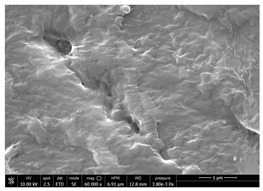

**Table 9:** Surface energy of substrates uncoated and coated with chitin + OLA. For each column of results, the same letter means that values were not significantly different (confidence level 95%).

Samples	Surface Energy [mN/m]	Dispersive [mN/m]	Polar [mN/m]
PHBV/PBS	40.8 ± 0.2 ^a^	32.1 ±0.1 ^a^	8.7 ± 0.1 ^a^
PHBV/PBS + CN fungi 3 h 1.5% + OLA	51.5 ± 0.4 ^b^	32.1 ± 0.2 ^a^	19.4 ± 0.2 ^b^
PBS	36.6 ± 0.2 ^c^	36.0 ± 0.2 ^b^	0.61 ± 0.1 ^c^
PBS + CN fungi 3 h 1.5% + OLA	55.6 ± 0.4 ^d^	29.4 ± 0.2 ^c^	26.2 ± 0.2 ^d^
PBAT/PLA	34.1 ± 0.3 ^e^	27.9 ± 0.2 ^d^	6.2 ± 0.1 ^e^
PBAT/PLA + CN fungi 3 h 1.5% + OLA	57.3 ± 0.8 ^f^	35.2 ± 0.5 ^e^	22.1 ± 0.2 ^f^

**Table 10:** Results of antibacterial tests on films treated with chitin (Aldrich) and chitin (Aldrich) + OLA1350. For each column of results, the same letter means that values were not significantly different (confidence level 95%).

Sample	Recovery after 24 h Incubation (Cells/cm^2^)		Antibacterial Activity (R)	
	*E. coli*	*S. aureus*	*E. coli*	*S. aureus*
PHBV/PBS	15,600 ± 1800 ^a,b^	687 ± 72 ^a^	/	/
PBS	5938 ± 620 ^c^	156 ± 17 ^b,c^	/	/
PLA/PBAT	6130 ± 830 ^c^	325 ± 44 ^b^	/	/
PHBV/PBS + CN fungi 3 h 1.5%	12,500 ± 1400 ^b,d^	356 ± 40 ^b^	0.09	0.29
PHBV/PBS + CN shrimp 3 h 1.5% + OLA	6875 ± 730 ^c^	13 ± 2 ^c^	0.35	1.73
PHBV/PBS + CN fungi 3 h 1.5% + OLA	10,625 ± 1200 ^d^	58 ± 8 ^c^	0.16	1.08
PLA/PBAT + CN shrimp 3 h 1.5% + OLA	350 ± 40 ^e^	38 ± 5 ^c^	1.25	0.93
PBS + CN shrimp 3 h 1.5% + OLA	0.33 ± 0.05 ^e^	3 ± 0.4 ^c^	4.25	2.79
PBS + OLA	17,500 ± 2000 ^a^	1875 ± 200 ^d^	0	0

**Table 11:** Results of biodegradability tests.

Sample	Absolute % Loss	Days
PBAT/PLA + CN fungi 3 h 1.5% + OLA	95.8 (Industrial composting)	135
PBSA/PHBV + CN fungi 3 h 1.5% + OLA	95.7 (Home composting)	255

## Data Availability

Not applicable.
